# The future of antibiotics

**DOI:** 10.1186/cc13948

**Published:** 2014-06-27

**Authors:** Brad Spellberg

**Affiliations:** 1 Division of General Internal Medicine, Harbor-University of California at Los Angeles (UCLA) Medical Center, 1124 West Carson St, Torrance, CA 90502, USA; 2 Los Angeles Biomedical Research Institute, 1124 West Carson St, Torrance, CA 90502, USA

## Abstract

Antibiotic resistance continues to spread even as society is experiencing a market failure of new antibiotic research and development (R&D). Scientific, economic, and regulatory barriers all contribute to the antibiotic market failure. Scientific solutions to rekindle R&D include finding new screening strategies to identify novel antibiotic scaffolds and transforming the way we think about treating infections, such that the goal is to disarm the pathogen without killing it or modulate the host response to the organism without targeting the organism for destruction. Future economic strategies are likely to focus on ‘push’ incentives offered by public-private partnerships as well as increasing pricing by focusing development on areas of high unmet need. Such strategies can also help protect new antibiotics from overuse after marketing. Regulatory reform is needed to re-establish feasible and meaningful traditional antibiotic pathways, to create novel limited-use pathways that focus on highly resistant infections, and to harmonize regulatory standards across nations. We need new antibiotics with which to treat our patients. But we also need to protect those new antibiotics from misuse when they become available. If we want to break the cycle of resistance and change the current landscape, disruptive approaches that challenge long-standing dogma will be needed.

## Review

### Introduction

It is difficult for modern physicians to appreciate the impact that the sudden availability of antibiotics had on the practice of medicine in the 1930s and 1940s [1]. Before antibiotics, physicians had little meaningful therapeutics with which to alter the course of their patients’ illnesses [2]. Then, suddenly, with the appearance of sulfanilamide in late 1936, followed by penicillin in 1942, cures came to be expected. As one eyewitness wrote, ‘The crossing of the historic watershed could be felt at the time. One day we could not save lives, or hardly any lives; on the very next day we could do so across a wide spectrum of diseases’ [3].

Indeed, the absolute reductions in mortality afforded by antibiotics are virtually unparalleled in the annals of medical pharmacotherapy. Conservative estimates of the absolute reductions in death mediated by antibiotic therapy include 25% for community-acquired pneumonia (CAP), 30% for nosocomial pneumonia, 75% for endocarditis, and 60% for meningeal or cerebral infections [4]. Even cellulitis, which is very rarely fatal in the modern era, mediated an 11% mortality in the pre-antibiotic era [5], and this rate is similar to the mortality of myocardial infarction in the placebo arm of the Second International Study of Infarct Survival study published in 1988 [6]. Furthermore, the absolute reduction in death from cellulitis mediated by antibiotics was more than 10% [5], as compared with a 3% reduction in death from myocardial infarction mediated by aspirin or streptokinase [6]. The ability to cure infections opened up entirely new fields in medicine, such as critical care medicine (for example, ventilators and central venous catheters), complex surgery, care of premature neonates, organ transplantation, and cancer chemotherapy.

Perhaps it is not surprising that the availability of such powerful weapons against disease quickly led to hubris. As early as 1948, one expert expressed ‘optimism’ that ‘bacterial diseases have been brought under control’ [7]. By 1962, a Nobel laureate pontificated that ‘one can think of the middle of the 20th century as the end of one of the most important social revolutions in history, the virtual elimination of the infectious diseases as a significant factor in social life’ [8]. The hubris continued through the 1980s [9], before the rise of antibiotic resistance began to bring us back to reality. During these decades of hubris, the medical community failed to understand that microbes have been waging war among themselves with antibiotics, and creating resistance mechanisms to defeat antibiotics, for more than two billion years [1,10]. We will never ‘defeat’ microbes with antibiotics. There is no ‘endgame’ - resistance is inevitable.

Nor is recognition of the threat of antibiotic resistance new, despite our failure to act effectively to confront the threat. Fifty years ago, a gathering of legends held a symposium focusing on the lack of new antibiotics that could deal with rising rates of resistant pathogens [11]. Indeed, as far back as 1945, Fleming himself, discoverer of penicillin, warned the medical community that our abuses of penicillin (and, by extrapolation, subsequent antibiotics) would surely lead to an inexorable rise in resistance, which ultimately would prove fatal for our patients [12]. ‘In such cases’, he said, ‘the thoughtless person playing with penicillin is morally responsible for the death of the man who finally succumbs to infection with the penicillin-resistant organism. I hope this evil can be averted’ [12].

Sadly, it has not been, and we have not learned from our past. We expose our environment to more than 15 million kilograms of antibiotics every year in the US alone [13]. This staggering degree of environmental contamination has, predictably, led to an inexorable rise in resistance rates, even as our research and development (R&D) efforts to develop new antibiotics have waned. Most pharmaceutical companies have abandoned the discovery and development of new antibiotics [14-16]. As a result, over the last 30 years, there has been a 90% decline in new approvals of systemic antibiotics by the US Food and Drug Administration (FDA) [10,17]. If we want to reverse these trends and facilitate new approaches to overcoming resistance, we must first understand the forces responsible for them.

### Causes of the antibiotic market failure

**Figure 1:**
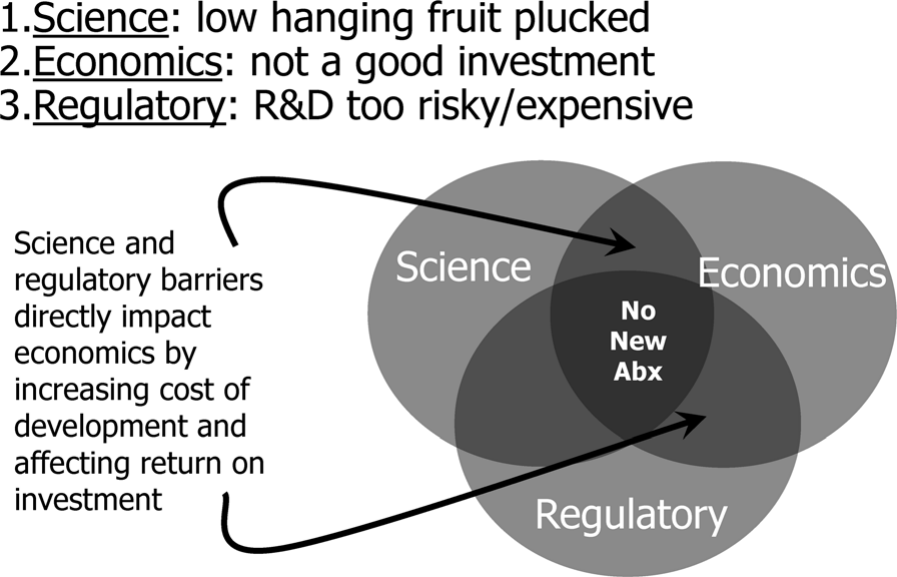
**Factors contributing to the antibiotic crisis.** Abx, antibiotics; R&D, research and development.

There are three primary causes of the antibiotic market failure, each of which interacts with, and exacerbates, the others (Figure 1).

### Scientific

More than 140 antibiotics have been developed for use in humans over the past 80 years [1]. Thus, we face considerable scientific barriers to discovering the next generation of antibiotics because the low-hanging fruit has been plucked. Using the same screening methodologies and the same chemical libraries tends to identify the same lead scaffolds over and over again [4,18,19]. Scientific complexity of discovery methodologies must therefore increase, which results in increasingly risky, time-consuming, and expensive discovery programs just at a time when economics and regulatory forces are converging to make antibiotics a poor vehicle for R&D investment. Furthermore, the ‘brain drain’ of expertise resulting from the systematic dismantling of antibacterial discovery programs at major pharmaceutical programs has exacerbated the difficulty in overcoming scientific complexities for new discovery.

These scientific complexities are further compounded for discovery of antibacterial agents targeting Gram-negative bacilli because of the unique biology of the Gram-negative cellular structure [19]. The lipid-rich membrane bilayer that envelops the cell wall creates unique physiochemical barriers to antibacterial penetration into the interior of the cells. Furthermore, porins and efflux pumps are ubiquitous among Gram-negative bacteria as a means to control nutrient and toxin influx and efflux and thus serve as natural resistance mechanisms for many antibacterial agents. These factors likely account for the lack of development of any new antibacterial classes for Gram-negative bacteria for more than 45 years (since nalidixic acid, the progenitor of the synthetic fluoroquinlones, was developed).

### Economic

Multiple economic factors make antibiotics less attractive for investment in R&D than other classes of drugs. For example, antibiotics are short-course therapies that cure their target diseases. Companies can make more money selling drugs that are taken every day for the rest of a patient’s life (for example, for hypertension, cholesterol, diabetes mellitus, acid reflux, arthritis, dementia, and HIV). Also, prices for antibiotics are typically not competitive with other drugs that have a high impact on morbidity and mortality (for example, cancer therapeutics). Small market sizes are further exacerbated by the appropriate principles of antibiotic stewardship, which lead thought leaders to advise judicious use when new antibiotics become available, such that sales of new antibiotics typically under-perform relative to expectations, particularly during the first years after market entry.

As a result of these and other market forces, a recent sophisticated study from the London School of Economics estimated that, at discovery, the net present value (NPV) of a new parenteral antibacterial agent was minus $50 million [20]. The NPV is a standard method that companies use to prioritize investment strategies that seeks to calculate today what the net value of a drug will be worth over the ensuing decades. It is calculated by incorporating cost input for R&D, time it will take to realize a return on investment, and future predicted revenues. By comparison, at discovery, the NPV for a new arthritis drug has been estimated to be positive $1 billion [14,16]. Given these economic realities, it is easy to understand why for-profit companies, which have a fiduciary responsibility to increase shareholder value, have increasingly shunted R&D money away from antibiotics and toward other drug types.

### Regulatory

For more than a decade, a shift in thinking at the FDA, particularly in the Office of Antimicrobials, has resulted in increasingly infeasible trial design requirements to enable new antibiotics to get approved for use in humans [4,17,21,22]. The reasons for this shift in thinking are complex, resulting in part from legitimate scientific and statistical concerns, but are driven to an irrational and dangerous extreme by the highly public and embarrassing post-marketing failure due to toxicity of the antibiotic, telithromycin [22,23]. In the end, statistical concerns have come to so thoroughly dominate considerations regarding trial standards that clinical reality and feasibility have been sacrificed.

Clear clinical trial guidances took many years to be released for trials of new antibiotics. When such guidances were released, they generally created trial conduct standards that were infeasible, nonsensical, or both [22,24]. Some experts even expressed doubt whether antibiotics were more effective than placebo for lethal infections, such as CAP [25,26]. Proposals were forwarded to force future antibiotic studies to use a placebo-controlled superiority design for the treatment of CAP - the disease that Osler referred to in 1901 as ‘the Captain of the Men of Death’ [27]. Such proposals were given serious credence and discussion and were discredited only after extensive and expensive dialogue and effort that took more than a year [25].

Other specific examples of unreasonable and damaging elements to new trial standards included banning any pre-study antibiotics from being administered to patients who were going to be enrolled in antibiotic clinical trials; this eliminated the possibility of enrolling any patients who were seriously ill. At the same time, studies were required to administer multiple days of intravenous therapy in hospital for diseases such as pneumonia, urinary tract infections, and intra-abdominal infections; this eliminated the possibility of enrolling any patients who were not seriously ill. Thus, there were few patients left to enroll.

New requirements that patients be considered evaluable for efficacy only if an etiologic bacterium was identified resulted in doubling or tripling of sample sizes for pneumonia studies. Non-inferiority margins shrunk because of arbitrary mathematical manipulations that were used to ‘discount’ the best guess of antibiotic treatment effect sizes for various diseases, further driving up sample sizes [22,25].

The cumulative effect of this ‘lost decade’ of debate, discussion, and deliberation was a substantial exacerbation of the risk, cost, and time it took to develop new antibiotics, just at the time when scientific challenges and other economic realities were having the same effect. The net result of these three converging forces, which fed off of one another, was a marked decrease in the number of companies, and number and experience of scientific experts, working in this space.

## Solutions to rekindle the pipeline

Given the above barriers, what solutions can be implemented to relieve the obstructions and rekindle antibiotic R&D in industry?

### Scientific

#### Finding new antibiotic scaffolds

Two broad methods may be useful to change the pool of antibiotics that can be discovered and developed [4]. The first is to change the substrate of the screen. Finding new natural sources for chemical substrate is a promising means to increase the likelihood of discovery of novel chemical scaffolds that can then become the focus of iterative substitutions and modifications to optimize effects. Only a small minority of microbial life has ever been successfully cultured, and new culture methods or new biochemical, proteomic, or metabolomics studies of such microbes could yield entirely new scaffolds for future development [4]. Other experts have written about means to enrich chemical libraries for scaffolds that are more chemically appropriate to penetrate into and not get effluxed from bacteria [19].

A second scientific approach is, rather than modifying the substrate of the screen, to modify the screening methodology to make the screening conditions more physiology relevant [28]. Traditional screens have used rich media to support robust microbial growth. However, the host environment is hostile to microbes as a result of both innate (for example, fever, phagocytes, complement, and pH changes) and adaptive (for example, antibody and cell-mediated immunity) host defense elements as well as nutrient restriction that is actively mediated by host factors (for example, trace metal and carbon source sequestration). It is likely that screening in media with more physiological pH or trace nutrient levels, with altered carbon sources [28], or with host defense constituents (for example, serum) in place would result in the identification of different scaffolds as promising therapies even from the same chemical libraries.

#### Transforming the goal of therapy

An even more transformative approach is to fundamentally change the way we think of disease, and to begin to try to treat infections by disarming pathogens or blunting excessive host response, rather than by trying to kill microbes [13]. Clinical disease is the outcome of interactions between microbe and host and may result as much (or more) from the host response to the microbe as from the microbe itself [29]. For example, treatment of *Acinetobacter baumannii* with a novel experimental antibiotic that blocks the rate-limiting step of lipopolysaccharide biosynthesis did not kill the bacteria but rendered them incapable of causing disease in mice [30]. Targeting virulence factors for neutralization, rather than targeting organisms for destruction, should exert substantially less selective pressure to drive resistance and is a promising way to approach new therapeutic development for infections [13].

Finally, direct targeting of the host, rather than the microbe, should also exert minimal selective pressure and can allow the host to minimize damage caused by invading microbes even if organisms are present [13]. Direct modulation of host inflammatory receptors, administration of sequestering agents that block access of microbes to essential host nutrients, passively starving the microbes and thus preventing replication, and use of probiotics to occupy ecological niches and compete with microbes are promising future strategies that could be useful to treat infections [13].

### Economic

Economic incentives are needed to rekindle the antibiotic R&D pipeline. Some help has already been provided to companies via passage into law of the Generating Antibiotic Incentives Now (GAIN) component of the FDA Safety and Innovation Act in 2012. The most direct economic support for antibacterial agents provided by GAIN is a maximum of 5-year extension of Hatch-Waxman ‘data exclusivity’, which helps prevent generic copies of drugs from reaching the market [31]. It is important to underscore that data exclusivity is distinct from, and runs concurrent with, patent time. Thus, drugs that reach the market with at least 10 years of patent life left will receive no exclusivity benefit from the GAIN Act [31]. However, for drugs that have little to no patent life left, GAIN creates serious financial incentive for development by restoring the potential for up to 10 years of exclusivity without generic competition.

Much of the previous attention on economic incentives has focused on ‘pull’ mechanisms, which are effective after a drug reaches approval [4,20,32,33]. Such mechanisms include extension of exclusivity, as discussed above, or the creation of ‘prizes’, guaranteed markets, or other downstream monetary rewards that kick in after approval. However, economic modeling has demonstrated that pull incentives are highly inefficient because of the effects of time discounting (a standard economic practice in which the value of future money is reduced by a set rate per year to adjust for risk and inflation) [20,31]. For example, a billion dollar prize pull incentive can be eroded to below $50 million in present value by time discounting [31]. Much more efficient, and more impactful at changing the NPV calculation which tends to dominate investment decisions in industry, are ‘push’ incentives that act during the discovery and development phase [31]. Such incentives include grants, contracts, and tax credits. Because they act early after discovery and during development, they are subject to far less time discounting than are pull incentives.

One effective means of distributing push economic incentives is to increase emphasis on public-private partnerships (PPPs) to rekindle the antibiotic R&D pipeline. Space limits preclude a thorough discussion of PPP structures and functions, but previous literature on these points is available [4,22,34]. Existing government PPP programs that focus on antibiotics already exist, principally through the Innovative Medicines Initiative’s New Drugs for Bad Bugs (ND4BB) program in the European Union, the Biodefense Advanced Research and Development Agency and National Institutes of Allergy and Infectious Diseases in the US Department of Health and Human Services, and to some extent in several agencies with the US Department of Defense. These programs have already awarded many hundreds of millions of dollars in grants or contracts to companies, both large and small, that are attempting to develop important new antibiotic candidates. They have served as the life support for antibiotic R&D over the last several years, and these programs need to be strengthened and continued.

An advantage of the PPP mechanism, which requires both a vigorous private industry and strong and focused support from government, is the ability to ensure that R&D efforts are aligned with unmet need. Traditional drug development programs in industry have focused on the largest markets, such as skin infections and CAP, since they are perceived to bring the largest return on investment and to be the easiest trials to complete. Thus, a plethora of new treatments for bacterial skin infections have become available in the last decade, even though there is little unmet need for such therapies. Even worse, when broad-spectrum antibiotics that can be used to treat lethal Gram-negative infections are developed to treat skin infections or even pneumococcal pneumonia, the drugs are wasted post-marketing on such diseases when numerous other, more narrow-spectrum therapies would suffice. Thus, we need to begin to align drug development with unmet need, to ensure that the drugs we need to save lives are being developed, and also to protect those drugs from overuse post-approval.

Ironically, the rise of highly resistant Gram-negative bacterial infections has created a new opportunity for an economic incentive: pricing. Pricing is the one ‘pull’ incentive that is likely to be highly effective at stimulating new industry R&D in the antibacterial space. If a new antibiotic is developed to treat lethal infections caused by resistant bacteria with limited to no alternative therapy, a marked pricing premium can be charged. As a result, a large market can result even if the number of cases is relatively small. For example, economic modeling indicates that a pathogen-specific therapy to treat carbapenem-resistant *A. baumannii* infections could be priced well in excess of $10,000 per treatment course, and perhaps as much as $30,000 per treatment course, while still easily meeting standard cost-effective metrics [35]. Such a therapy would be attractive to invest in, would focus on major unmet need, and would be protected from overuse or misuse for more sensitive, easier to treat infections because of its high pricing.

### Regulatory

Three fundamental elements of reform regarding regulatory standards for new antibiotic development are needed. First, traditional non-inferiority designs must be feasible and relevant. The European Medicines Agency (EMA) has released recent guidances that meet these objectives and set logical, reasonable, and achievable standards for such studies [36,37]. In contrast, in the US, progress has been slow but is possibly accelerating. In 2012, Janet Woodcock, director of the Center for Drug Evaluation and Research at the FDA, announced that the FDA was going to ‘reboot’ its focus on antibiotic trials [17]. She acknowledged that there was a serious crisis of antibiotic resistance and that prior FDA approaches to antibiotic trials had contributed to the slowing of new antibiotic development. We await the release of updated trial guidances that focus on feasibility and scientific and clinical rigor such that meaningful non-inferiority trials can be conducted to enable important new therapies to become available for our patients [17].

Second, we need to focus on facilitating development of antibiotics that meet high-impact, unmet need. Several ideas have been forwarded for new regulatory paradigms that would facilitate focusing on unmet need, including the Limited Population Antibacterial Drug (LPAD) pathway proposed by the Infectious Diseases Society of America [38], and the PhRMA four-tiered approach to development [39]. In their four-tiered approach, the PhRMA Tier C is very similar to LPAD, calling for small trials, possibly historically controlled, focusing on lethal infections with limited alternative available therapy. Both the EMA and the FDA have indicated support for such novel approaches and have released draft guidances reflecting this support [36,40].

But sponsors too need to recognize the shifting landscape. It is no longer true that older, traditional entry indications, such as skin infections and CAP, are the least risky and least expensive ways to develop drugs. Crowded entry indications have become commodities markets, with numerous competitor antibiotics already available, driving down pricing and creating (appropriately) a minimal risk tolerance threshold at the regulatory level. Trials focused instead on highly resistant bacterial pathogens for which limited available therapy exists may well be less expensive and shorter to conduct and can be used to support premium pricing, as discussed above. Such a situation is favorable for our patients, as it supports focus on true unmet need, and also helps prevent misuse and overuse post-marketing, as discussed above. Feasibility of such studies will increase over time as rapid molecular diagnostic tests become available to support their conduct [41].

Third, there must be harmonization between US and European regulators and ideally regulators in other parts of the world as well. The FDA exists in a very different political and legal climate than other national regulatory agencies, and it is true that congressional or public advocacy pressure has been instrumental in causing the FDA to become hyper-defensive in its recent approach to antibiotic pathways [22,23]. Nevertheless, leadership that is strong enough to do the right thing is needed, even in the face of such concerns. The EMA has already set reasonable standards for clinical trial conduct, and the EMA and other regulatory agencies will not harmonize with approaches that are unreasonable or infeasible. A harmonized approach across nations ultimately will be required to facilitate new antibiotic availability in this era of global economy.

Scientific thought leaders must be prepared to help explain complex scientific and clinical concepts to the public and to political leaders in order to help bring a more reasoned, patient-centric, and clinically relevant approach to antibiotic development in the US, which ultimately will support trans-national harmonization of standards.

## Solutions to prevent emergence of resistance

While the primary focus of this article is on facilitating new antibacterial development, we must stop making the same mistake over and over again with respect to misuse and overuse of antibiotics. Resistance will occur to all new antibiotics developed. Thus, as we facilitate new antibiotic development, we must re-emphasize our core responsibility as physicians and members of our communities to help preserve and protect the precious, limited, and exhaustible resource of antibiotics. Summaries of novel, disruptive approaches to enhancing infection prevention, disinfection, and decontamination; use of rapid diagnostics to enhance stewardship; and other improvements in antibiotic stewardship have been recently published [4,13,34].

The struggle with microbes will not end with new antibiotic availability. Indeed, it will never end. We bear great responsibility to protect the ‘awesome power’ of antibiotics that our forebears in medicine bestowed upon us. To prescribe a powerful new antibiotic is easy. To protect it is hard. But if we want to break the cycle and stop repeating the same mistakes of the past over and over, we must learn how to not use and abuse new antibiotics.

## Conclusions

Highly accomplished clinician-scientists who experienced the before and after of the availability of antibiotics described their effect as being ‘almost beyond belief’ [2] and ‘an awesome acquisition of power’ [3]. Unfortunately, the crisis of antibiotic resistance threatens to dissipate that power. The pipeline has dried up and resistance continues to worsen year by year. As a result, a national survey of infectious diseases specialists in 2012 revealed that more than half of them had been confronted with pan-resistant bacterial infection during the previous year [42]. This problem will continue to grow worse unless we fundamentally change the way our society deals with the discovery, development, use, and protection of these life-saving drugs.

### Note

This article is part of a series on *Antibiotic resistance in the ICU*, edited by Steven Opal. Other articles in this series can be found at http://ccforum.com/series/antibioticresistance.

## Abbreviations

CAP: Community-acquired pneumonia; EMA: European Medicines Agency; FDA: US Food and Drug Administration; GAIN: Generating Antibiotic Incentives Now; LPAD: Limited Population Antibacterial Drug; NPV: Net present value; PPP: Public-private partnership; R&D: Research and development.

## Competing interests

Within the last year, BS’s employer has received consulting fees on his behalf from GlaxoSmithKline (Uxbridge, Middlesex, UK), Meiji Seika Pharma Co., Ltd (Tokyo, Japan), Cardeas Pharma (Seattle, WA, USA), Affinium Pharmaceuticals (Austin, TX, USA), aRigen Pharmaceuticals (Tokyo, Japan), and Synthetic Biologics (Rockville, MD, USA) and grants or contracts for basic science or clinical research from Cubist (Lexington, MA, USA), Pfizer (New York, NY, USA), and Bristol-Myers Squibb Company (Princeton, NJ, USA).
